# Drug Resistance Reversal Potential of Nanoparticles/Nanocomposites via Antibiotic’s Potentiation in Multi Drug Resistant *P. aeruginosa*

**DOI:** 10.3390/nano12010117

**Published:** 2021-12-30

**Authors:** Pratima Pandey, Rajashree Sahoo, Khusbu Singh, Sanghamitra Pati, Jose Mathew, Avinash Chandra Pandey, Rajni Kant, Ihn Han, Eun-Ha Choi, Gaurav Raj Dwivedi, Dharmendra K. Yadav

**Affiliations:** 1 Department of Biotechnology, Bundelkhand University, Jhansi 284128, India; bajpai.pratima@rediffmail.com; 2 Nanotechnology Application Centre, University of Allahabad, Allahabad 211002, India; 3 Microbiology Department, ICMR-Regional Medical Research Centre, Bhubaneshwar 751023, India; rajashree.rainy777@gmail.com (R.S.); singh.khusbu253@gmail.com (K.S.); drsanghamitra12@gmail.com (S.P.); jose_feb_25@yahoo.co.in (J.M.); acpandey@iuac.res.in (A.C.P.); 4 Microbiology Department, ICMR-Regional Medical Research Centre, Gorakhpur 273013, India; rajnikant.srivastava@gmail.com; 5 Plasma Bioscience Research Center, Applied Plasma Medicine Center, Department of Electrical & Bio-logical Physics, Kwangwoon University, Seoul 01897, Korea; hanihn@kw.ac.kr; 6 College of Pharmacy, Gachon University, Hambakmoeiro 191, Yeonsu-gu, Incheon City 406-799, Korea

**Keywords:** nanoparticle, nanocomposite, drug resistance reversal, MIC, graphene oxide–chitosan

## Abstract

Bacteria employ numerous resistance mechanisms against structurally distinct drugs by the process of multidrug resistance. A study was planned to discover the antibacterial potential of a graphene oxide nanosheet (GO), a graphene oxide–zinc oxide nanocomposite (GO/ZnO), a graphene oxide-chitosan nanocomposite (GO–CS), a zinc oxide decorated graphene oxide–chitosan nanocomposite (GO–CS/ZnO), and zinc oxide nanoparticles (ZnO) alone and in a blend with antibiotics against a PS-2 isolate of *Pseudomonas aeruginosa*. These nanocomposites reduced the MIC of tetracycline (TET) from 16 folds to 64 folds against a multidrug-resistant clinical isolate. Efflux pumps were interfered, as evident by an ethidium bromide synergy study with nanocomposites, as well as inhibiting biofilm synthesis. These nanoparticles/nanocomposites also decreased the mutant prevention concentration (MPC) of TET. To the best of our knowledge, this is the first report on nanomaterials as a synergistic agent via inhibition of efflux and biofilm synthesis.

## 1. Introduction

Disease is a condition of comprehensive physical abnormality and physiological disorder (WHO). Diseases can be categorized as infectious or non-infectious based on the mode of transmission. Communicable diseases are the major causes of illness and death worldwide [1]. Antibiotics are treated as the marvel of drugs to fight against microbes, but the rampant and unaware use of drugs with limited knowledge of targets, lack of novel antibiotics and vaccines have increased the level of resistance in bacterial pathogens [2,3,4]. The continuous burden of antibiotics on microbes helps in the evolvement of single drug-resistant, multidrug-resistant, and total drug-resistant bacteria [4,5,6].

Bacterial infections refer to the proliferation of a harmful strain on the surface or inside of the host body. They can infect any part of the body. Some Gram-positive and Gram-negative bacteria cause many of the deadliest diseases. The Enterobacteriaceae family, *Pseudomonas*, *Acinetobacter*, *Mycobacterium*, *Helicobacter*, and *Treponema* spp., are reported as life-threatening disease-causing bacteria [4,7,8]. Some of the common diseases caused by these bacteria are urinary tract infections, gastroenteritis, sepsis, food poisoning, lung infections, cystic fibrosis, wound infections, plague, and tuberculosis [4,8,9,10,11].

In a 2017 report, WHO identified a list of a dozen antibiotic-resistant bacteria (Dirty dozen) based on the severity of infection, treatment cost, and need for novel antibiotics [5,8]. These dozen bacteria were further placed under critical, high, and medium priority groups. *Pseudomonas aeruginosa*, *Acenetobacter baumannii*, and *Enterobacteriaceae* members were categorized into critical groups [7,12]. To date, antibiotics are the only major treatment option to control critical pathogens [2,3,13]. The onset of antibiotic resistance enhances the loss of the antibiotic ability to inhibit these bacterial pathogens, and these resistant bacteria multiply in the presence of antibiotics [7,14]. By certain mechanisms, the bacteria are developing resistance against antibacterial drugs. Biofilm matrices are the three-dimensional cumulative collection of microbes in which cells frequently stick to the surface and attach within a meshwork of extracellular polymeric substances (EPSs), mainly consisting of polysaccharides, some proteins, and extracellular nucleic acids. Bacterial biofilm is a survival mechanism that delivers the capability to resist environmental strain and drugs/antibiotics, plus the least metabolic action [15]. Efflux pump is a biological pump that expels the antimicrobial compounds from the cell to the outside; alongside this, the downregulation of porin channels prevents the entry of drugs to the cells, which ultimately develops bacterial drug resistance [16,17,18]. The β-lactamase enzyme is responsible for the breakdown of the β-lactam ring in a β-lactam group of antibiotics. Biofilm, efflux pump, porins, and β-lactamase are four major mechanisms responsible for multidrug resistance (MDR) in bacteria [17,18,19]. Insufficient antibiotics/drugs choice exists to treat these pathogens, and several agents (polymyxins/aminoglycosides/tigecycline) are reported with noteworthy toxicities. Few antibiotics are now used in combination, such as ceftolozen/tazobactam, ceftazidime/avibactam, and meropenem/vaborbactam [20,21]. Nanoparticles (1–100 nm) are supposed to be one of the emerging warheads to counter bacterial drug resistance [22,23].

Because of the above problem posed by the critical superbug *P. aeruginosa*, the absence of proper therapy, and past ventures on the reversal of drug resistance and antibacterial activity, the current study assesses nanomaterials as a novel treatment system against MDR *P. aeruginosa*. Initially, the antibacterial activity was evaluated, and efforts were also made to investigate the mechanism of action of nanomaterials against MDR *P. aeruginosa*. The overall experimentation in this study involved: design and synthesis of nanomaterials, mechanism of drug resistance in critical superbug *P. aeruginosa*, evaluation of the nanocomposites as an anti-pseudomonal agent, evaluation of nanomaterials as a drug-resistant reversal agent, study of the mode of action of nanomaterials, and study of the “druggability” of nanomaterials.

## 2. Materials and Methods

Potassium hydroxide, zinc acetate dihydrate, sodium nitrate, sulphuric acid, hydrochloric acid, methyl alcohol, and ethyl alcohol were obtained from Merk India Ltd. (grade AR). High molecular weight deacetylated chitin (Chitosan powder (CS)), N-(3-Dimethylaminopropyl)-N′-ethylcarbodiimide hydrochloride (EDC hydrochloride), and NHS (N-hydroxysuccinimide) were purchased from Sisco Research Laboratories Pvt Ltd. (Mumbai, India) Graphene oxide nanosheet was purchased from Reinste Nano Ventures (Noida, India). Potassium dihydrogen phosphate (KH_2_PO_4_) and other chemicals used during the synthesis were obtained from Merck Ltd., SRL Pvt. Ltd. (Mumbai, India). Double distilled water was used during the synthesis process.

The synthesized nanomaterials were investigated by X-ray diffractometer (XRD) and Scanning Electron Microscope (SEM) for structural properties and Fourier Transform Infrared Spectroscopy (FTIR) to examine the surface of functional groups. The FTIR analysis was performed on FTIR spectroscopy (Perkin Elmer FTIR Spectrum, BX-II, USA). The XRD analysis was performed on X-ray diffractometer (Rigaku D/max 2200 PC, Japan) using Cu Kα radiation between 10–80° intervals. The surface morphology was investigated by field emission scanning electron microscopy (MIRA II LMH from TESCAN, with a resolution of 1.5 nm at 30 kV, Czech Republic).

### 2.1. Protocol for Synthesis of Graphene Oxide and Zinc Oxide Nanocomposite (GO/ZnO)

The synthesis protocol of graphene oxide and zinc oxide nanocomposite was as per Chowdhuri et al., with minor modifications [23]. Solution X was prepared by adding 0.20 g of graphene oxide suspension in 20 mL water (double distilled water). Solution X was sonicated and kept 20 min for uniform mixing. At same time, solution Y was prepared by adding 1.4 g of zinc acetate dihydrate in 60 mL methyl alcohol. Gradually, solution X was mixed into solution Y, then the final reaction mixture was sonicated to agitate the particles and kept 25 min for uniform mixing. After that, the reaction mixture was shifted into a 250 mL Erlenmeyer flask. The prepared potassium hydroxide solution was added steadily into the reaction mixture of solution X and Y in a 250 mL Erlenmeyer flask, and the reaction was continued to 8 h at 60 °C. The KOH solution was ready by mixing 0.6 g of KOH in 20 mL of methyl alcohol. The precipitates were collected by centrifugation at 10,000 rpm for 15 min and washed four times by a mixture of methyl alcohol–water to eliminate contamination.

### 2.2. Protocol for Synthesis of Graphene Oxide and Chitosan (GO-CS)

The synthesis protocol of the graphene oxide and chitosan nanocomposite was as per Chowdhuri et al., with minor modifications [23]. First, the chitosan solution was prepared by adding 0.25 g of chitosan (CS) in 250 mL 1% acetic acid solution and kept reaction mixture for uniform mixing until 1.5 h. In the second step, the graphene oxide reaction mixture was prepared by adding 0.375 g of GO nanosheet in 50 mL water (double distilled water). After that, a total of 0.375g, N-(3-dimethylaminopropyl-N-ethylcarbodiimide) hydrochloride (EDC) was added. HCl and 0.25 g of NHS were added into the prepared GO suspension and kept the reaction mixture for uniform mixing until 3.5 h. In the final step of the synthesis of the GO–CS nanocomposite, the GO reaction mixture was added into the chitosan solution very gently and kept them for uniform mixing until for 10 h. The final product was collected by centrifugation at 10,000 rpm for 15 min and dried at 40 °C under a vacuum oven.

### 2.3. Protocol for Synthesis of Graphene Oxide, Chitosan, and Zinc Oxide Nanocomposite (GO–CS/ZnO)

For the synthesis of GO–CS/ZnO nanocomposite, solution X was prepared by adding already prepared 0.2 g GO–CS in 20 mL water (double distilled water). Solution X was sonicated to agitate the particles and kept 15 min for uniform mixing. At the same time, solution Y was prepared by adding 1.4 g of zinc acetate dehydrate in 60 mL methyl alcohol. Solution X was added gently to solution Y, sonicated, and stirred for 20 min. After that, the reaction mixture was shifted in 100 mL Erlenmeyer flask. The prepared potassium hydroxide solution was added steadily in reaction mixture of solution X and Y in 100 mL Erlenmeyer flask, and the reaction was continued to 10 h at 60 °C. The KOH solution was made by mixing 0.6 g of KOH in 20 mL of methyl alcohol. The final product was collected by centrifugation at 10,000 rpm for 15 min and washed four times by a mixture of methyl alcohol–water to eliminate contamination and dried at 40 °C under a vacuum oven.

### 2.4. Protocol for Synthesis of ZnO Nanoparticles

The synthesis protocol of zinc oxide nanoparticles was as per Singh et al., with minor modifications [24]. Zinc oxide nanoparticles were prepared by adding 1.4 g of zinc acetate dihydrate, which was mixed in 60 mL methyl alcohol. The prepared potassium hydroxide solution was added steadily into to the above solution and the reaction mixture was kept for uniform mixing until 10 h at 50 °C. KOH solution was made by mixing 0.6 g of KOH in 20 mL of methyl alcohol. The final product as a white precipitate was collected by centrifugation at 10,000 rpm for 10 min and washed four times by a mixture of methyl alcohol–water to eliminate contamination and dried at 60 °C under a vacuum oven.

### 2.5. Procurement of Clinical Bacterial Isolates

MDR clinical isolates, namely PS-2, PS-3, and PS-11, were procured from the Regional Medical Research Centre, Bhubaneswar, repository.

### 2.6. Disc Diffusion Assay (DDA)/Kirby-Bauer Antibiotic Test

This study was conducted as per the agar diffusion test (modified Kirby–Bauer antibiotic testing), a test used for the antibiotic sensitivity of bacteria [25]. The sensitivity profiling of the graphene oxide nanosheet (GO), graphene oxide–zinc oxide nanocomposite (GO/ZnO), graphene oxide–chitosan nanocomposite (GO–CS), zinc oxide decorated graphene oxide–chitosan nanocomposite (GO–CS/ZnO), and zinc oxide nanoparticles (ZnO) was carried out by impregnating antibiotic discs on Muller–Hinton Agar (MHA) plates. Overnight grown cultures were diluted up to 0.5 McFarland units and a lawn culture was prepared. The incubation of plates was performed at 37 °C for 24 h and the zone of inhibition was measured (in mm).

### 2.7. Broth Dilution Assay

In the Mueller–Hinton broth (MHB), the minimum inhibitory concentrations (MICs) were determined by using 96-well microtiter plates following the Clinical and Laboratory Standards Institute guidelines for broth microdilution [26]. In this test, the desired concentration of antibiotic (10 μg/μL), by making a dilution from a stock solution and taking a 96-well plate, was performed, and the labeling was conducted. A total of 150 μL of MHB broth was added to each well. After being properly mixed, the serial dilution process was continued up to the 10th well (1600 to 1.56 mg/L). The 24 h grown bacterial culture was diluted to find out the appropriate inoculum size for the standard equivalent to 0.5 McFarland standards. An inoculum of 10 μL of 0.5 McFarland standards was dispensed in each well except in the negative control. The plate was incubated at 37 °C for 24 h. After the reading was taken, the result was interpreted as per CLSI guidelines.

### 2.8. Synergy Studies with Imipenem/Ethylene Diamine Tetraacetic Acid (EDTA)

For the metallo-β-lactamase (MBL) test, a 24 h fresh bacterial culture was used. For this, a 0.5% McFarland standard was maintained. A sterile swab was soaked inside the bacterial culture tube. Then, the bacterial culture was spread on the Muller–Hinton Agar (MHA) plate. Sterile imipenem with or without Ethylene Diamine Tetraacetic Acid (EDTA) strip was placed on the plate and incubated for 24 h for observation. The synergistic effect of GO, GO/ZnO, GO–CS, GO–CS/ZnO, and ZnO with imipenem was studied along with EDTA added to imipenem as a positive control and imipenem only as a negative control [27]. A lawn culture of 0.5 McFarland unit was prepared by swabbing the overnight grown culture onto MHA plates and 10 µL of nanoparticle was added onto an impregnated imipenem disc.

### 2.9. Biofilm Formation/Inhibition Assay

This was conducted as per the prescribed protocol, which includes biofilm formation and the impact of inhibitors on biofilm synthesis [28,29]. A 1/100 dilution was performed for a 24 h grown culture. In 96-well plates, the addition of diluted bacterial culture in 96-well plates was carried out except in the negative control (only with 100 µL broth). GO, GO/ZnO, GO–CS, GO–CS/ZnO, and ZnO were added, and the plate was incubated for 48 h. Washing was conducted twice using saline water 0.7% saline (NaCl by inverting the plate). Incubation was conducted for 3 h, then crystal violet was added and again incubated for 20 min. Twice washing was again carried out by normal saline and acetic acid was added. Next, it was transferred into a new 96-well plate and scanned under an ELISA reader.

### 2.10. Ethidium Bromide–Agar Cartwheel Method

This method was adapted for the presumptive identification of multidrug-resistant bacterial isolates that overexpress efflux pump systems [6]. Different plates containing different concentrations of ethidium bromide were made. After solidification, a fresh bacterial culture was swabbed on the plate. The plates were subjected to incubation for 24 h. Then, they were observed under UV-transilluminator to check whether they flourish.

### 2.11. Combination Assay/Broth Checkerboard Method

This study was led using a broth checkerboard assay [30]. Pipetting of 150 µL of pH adjusted MHB was conducted to adjust the dilutions, such that each well had diverse concentrations of tetracycline (TET) and nanomaterials. The concentration of TET was varied between 12.5–1600 mg/L and for 3.25 to 200 mg/L for GO, GO/ZnO, GO–CS, GO–CS/ZnO, and ZnO. Documented results were given in terms of a) type of interaction, b) fold reduction, and c) fractional inhibitory concentration index (FICI). The FICI index depicts results in terms of the antagonism (>4.0) no interaction (0.5–4.0) and synergism (<0.5) [31].

### 2.12. Biofilm Inhibition Assay

For the biofilm inhibition assay, we made a 1/100 dilution of the 24 h culture. Then a 96-well plate was taken, and it was filled with the bacterial culture except for negative control. In the negative control, only 150 μL MHB broth was taken. Then, the addition of different inhibitors (GO, GO/ZnO, GO–CS, GO–CS/ZnO, and ZnO) as per the calculation was performed. After adding the inhibitors, we incubated the plate for 48 h. Next, plate was washed simply by inverting it. Then, we washed the plate with 0.7% saline (NaCl) twice and washed it by inverting the plate. Then, the plate was incubated for 3 h. Then, 1% crystal violet was added and incubated for 20 min. Then, the plate was washed with normal saline twice. Then, we added acetic acid. Next, we transferred this into a new 96-well plate and scanned it under an ELISA reader.

### 2.13. Efflux Pump Inhibition Assay

Resistance in EtBr is considered a marker of MDR mediated by efflux pump. The MIC test of EtBr was carried out by a broth dilution assay and the combination study was also performed by the method described above [30].

### 2.14. Drug Ability Study of Nanomaterials by Mutation Prevention Concentration Method (MPC)

MPC of TET was conducted using *P. aeruginosa* MTCC 741 as per the procedure of Heisig and Tschorny [32]. MPC of TET was performed individually or in the company of GO, GO/ZnO, GO–CS, GO–CS/ZnO, and ZnO at diverse concentrations (2× MIC, 4× MIC, 8× MIC, and 16× MIC). Outcomes were attained by dividing the total number of colonies after 48 h of incubation at 37 °C on the antibiotic-containing plate by the total number of colony-forming units plated.

## 3. Results

### 3.1. Chemistry

#### 3.1.1. FTIR (Fourier Transforms Infrared Spectroscopy) Study

**Figure 1:**
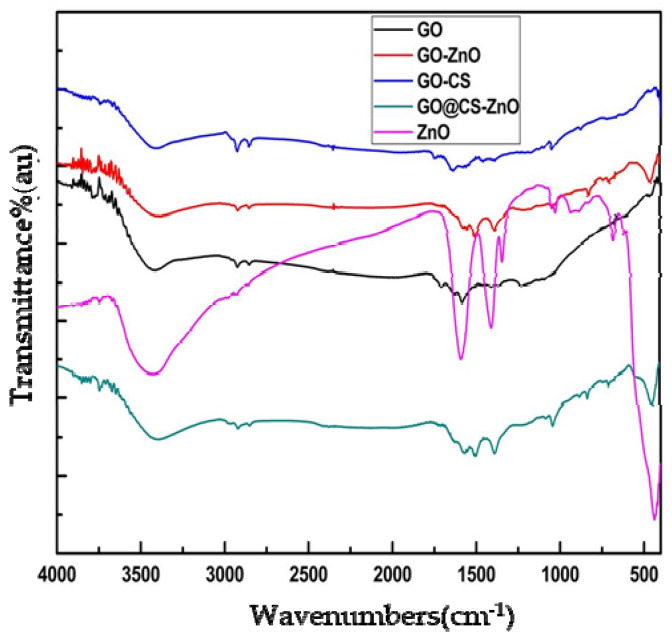
FTIR spectra of nanomaterials (GO, GO/ZnO, GO–CS, GO–CS/ZnO, and ZnO).

The presence of zinc oxide (ZnO), chitosan (CS), and graphene oxide (GO) separately in the synthesized nanocomposite GO–CS/ZnO was verified by using FTIR technique. The sharp peak at 454 cm^−1^ for zinc oxide (ZnO) stretching frequency verified that ZnO is efficiently binding both GO and GO–CS. The peculiar feature of GO in the FTIR spectra exemplifies absorption bands at 1723 cm^−1^ due to the C=O [33] stretch of the carboxylic group (COOH), at 1621 cm^−1^ for a stretch of C=C groups, at 1224 cm^−1^ for a stretch of C–OH [33], at 1043 cm^−1^ for a stretch of alkoxy C–O groups [33], and a very wide strong peak at 3415 cm^−1^ for O–H stretching frequencies. The appearance of peaks at 1094 cm^−1^ and 1394 cm^−1^ also shows the characteristic peaks for the stretching vibration frequency of the C–O–C and C–OH bond of GO [34]. The distinctive peaks of chitosan (CS) are at 3447, 2850, 1645, 1566, 1161, 1056 cm^−1^ [35]. The peak at the range of 2850 cm^−1^ indicates the effective formation of chitosan over the surface of GO. The appearance of peak at 459 cm^−1^ verified that ZnO was incorporated on GO and GO–CS, as shown in Figure 1.

#### 3.1.2. X-ray Diffraction (XRD) Analysis

**Figure 2:**
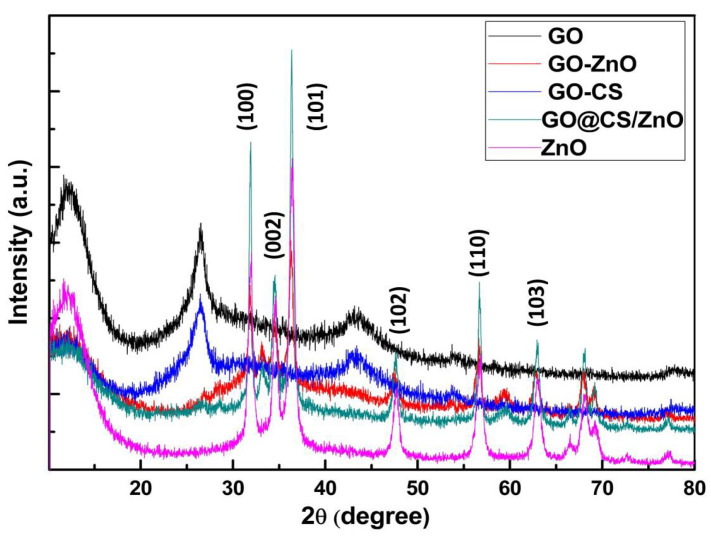
XRD patterns of GO, GO/ZnO, GO–CS, GO–CS/ZnO, and ZnO.

The presence of ZnO nanoparticles in the GO layer was verified by the XRD analysis shown in Figure 2. The characteristics peaks observed in order at 2*θ* = 31.87°, 34.55°, 36.42°, 47.66°, 56.64°, 62.92°, 68.01°, 69.20° are matched with the standard (JCPDS card no: 80-0074) and are in good agreement with the crystalline planes of ZnO nanoparticles (corresponding to (1 0 0), (0 0 2), (1 0 1), (1 0 2), (1 1 0), (1 0 3), (1 1 2), and (2 0 1)). When the ZnO nanoparticles were anchored on the surface of graphene oxide (GO), the characteristic peak of graphene oxide at 2θ = 110° vanished. The XRD pattern of the GO–Cs/ZnO nanocomposite showed a peak at 2θ = 14.540, 59.30, and a wide peak above 19.5°, which verified the presence of the amorphous state of chitosan.

#### 3.1.3. Surface Morphology Study

The surface morphology of GO–CS/ZnO was explored by scanning electron microscopy (SEM), shown in supplementary data (Figure S1). The micrograph of GO–CS/ZnO at 158.43 magnification highlighted the presence of the graphene oxide nanosheet and chitosan, and ZnO nanoparticles over and around the layers of the graphene sheet. The figure also showed the almost uniform decoration of ZnO nanoparticles over the surface of the graphene nanosheet with nearly spherical morphology.

### 3.2. Biological Evaluation

Initially, three clinical isolates of *P. aeruginosa* (PS-2, PS-3, and PS-11) were used, based on sensitivity/resistance profiling, Imipenem-EDTA synergy, biofilm synthesis, and agar cartwheel; PS-2 isolate was most resistant. PS-2 was resistant against ampicillin, tetracycline, cephalosporin, gentamycin, ciprofloxacin, chloramphenicol, and piperacillin. This confirms that the bacterial isolates were resistant against different types of structurally unrelated antibiotics (Table S1). Due to luxuriant growth on the high concentration of ethidium bromide, the synthesis of biofilm and production of metallo-β-lactamase make PS-2 more recalcitrant towards different antibiotics. It was also observed that clinical isolates were resistant to structurally and functionally different antibiotics. Among all clinical isolates, PS-2 isolate of *P. aeruginosa* was selected for further studies due to the high-level resistance towards different antibiotics, luxuriant growth on a high concentration of ethidium bromide, the highest production of biofilm, and production of metallo-β-lactamase.

#### 3.2.1. Antibacterial Potential of Nanomaterials

The antibacterial potential of nanomaterials was evaluated by a broth dilution assay. The MIC of the GO, GO/ZnO, GO–CS, GO–CS/ZnO, and ZnO ranges from 400–800 mg/L, which is shown in Table 1. Based on MIC, GO–CS/ZnO was found comparatively potent (MIC 400 mg/L), while nanomaterials (GO, GO/ZnO, GO–CS, and ZnO) were less effective (MIC 800 mg/L). Based on stringent activity criteria, these nanomaterials did not fall in the category of antibacterial agents.

#### 3.2.2. Antibacterial Potential of Nanomaterials in Combination with Antibiotic Tetracycline

In a combination study, tetracycline was used as a partner drug. The MIC of tetracycline was reduced from 16–64 folds in the presence of nanomaterials. Based on FICI, the nanomaterials interaction with tetracycline was synergistically shown in Table 1.

To understand the drug resistance reversal potential of nanomaterials (GO, GO/ZnO, GO–CS, GO–CS/ZnO, and ZnO), different modes of action studies were performed.

#### 3.2.3. MBL Inhibitory Study of Nanomaterials with Imipenem

**Figure 3:**
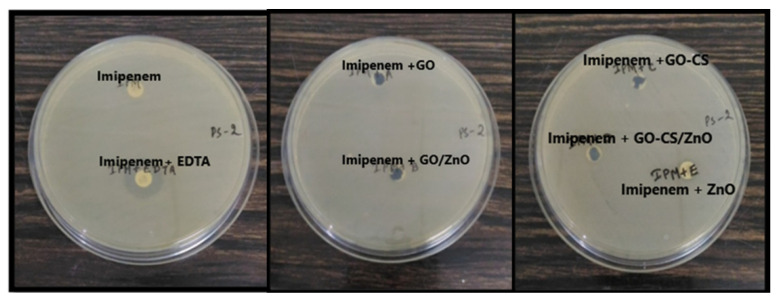
Interaction study of nanomaterials: imipenem and imipenem-EDTA (in mm).

To study the MBL inhibition property of nanomaterials, an imipenem-EDTA synergy study was performed. In this study, GO, GO/ZnO, GO–CS, GO–CS/ZnO, and ZnO did not inhibit the MBL as it is evident in a net zone of inhibition. However, imipenem plus EDTA showed a clear net zone of inhibition, while there was no zone of inhibition in either imipenem or imipenem and the nanomaterials, as shown in Figure 3.

In Figure 3, it can be seen that the imipenem disc was the negative control as we know that PS-2 is resistant to imipenem and imipenem with EDTA was the positive control as EDTA acts as a chelating agent of the metallo-β-lactamase enzyme. It was concluded that these nanoparticles/nanocomposites were not able to inhibit the metallo-β-lactamases enzyme produced by the PS-2, as it did not show any zone of inhibition with imipenem.

#### 3.2.4. Biofilm Inhibitory Potentials of Nanomaterials

**Figure 4:**
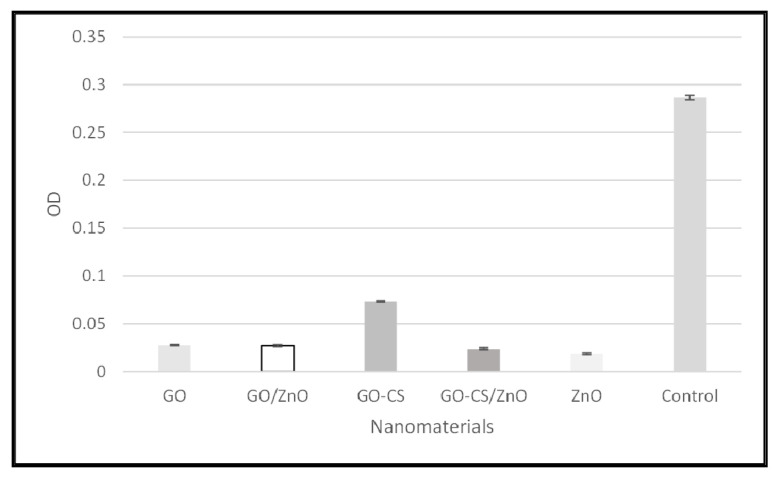
Biofilm inhibitory potential of nanomaterials.

In this study, all the nanomaterials inhibited the biofilm synthesis as the OD is comparatively very low in the presence of nanomaterials, which is shown in Figure 4.

#### 3.2.5. Ethidium Bromide Synergy Potential of Nanomaterials

Alone the MIC of ethidium bromide is 1600 mg/L, which was reduced up to 16 fold in the presence of nanomaterials, as shown in Table 2.

#### 3.2.6. Mutant Prevention Concentration (MPC) of Nanomaterials

All the nanomaterials reduced the MPC of TET, which increases the relevance of these as the right agent for therapeutic purposes, as shown in Table 3.

## 4. Discussion

After the inception of the antibiotic’s penicillin and streptomycin, chemotherapy was revitalized [3,14]. Antibiotics renovated the chemotherapy industry and in the 1980s contagious diseases were considered diseases of the past with a high life expectancy [13,36,37,38]. The unrestrained applications of antibiotics have created several bacteria, including *P. aeruginosa*, to gain resistance to multiple drugs [39,40,41]. The discovery of the novel antibiotic teixobactin offered efficacy against many drug-resistant bacteria, but there is no new antibiotic against many MDR bacteria [42,43]. The efforts of the FDA, the Infectious Diseases Society of America (IDSA), and the European Medicines Agency will start to recognize new antibiotics in the upcoming future [44]. Based on all findings, it may be concluded that these nanoparticles boost the hope to explore these as drug resistance reversal agents.

The carbapenem group of antibiotics (meropenem and imipenem) were reported as the last option against Gram-negative pathogens [12,20,38,42,45,46]. The clinical isolates of *P. aeruginosa* (PS-2, PS-3, and PS-11) were the producer of MBL, which hydrolyze the carbapenem group of antibiotics. There are several reports according to which MBL enzymes are the key mechanism of resistance towards the above antibiotics [12,15,20,45,46]. Biofilms are the extracellular matrix that is responsible for the protection of cells from different stresses, including antibiotic stress [15,29,39,44]. Biofilm-producing microbes have also been known for their resistance to a range of antimicrobial agents, including clinically relevant antibiotics [15,29,39,44,47]. Overexpressed efflux pump mechanism is evident from the luxuriant growth on the high concentration of Et-Br, which is evident by a higher level of fluorescence under the UV-transilluminator. Efflux pumps are supposed to be the key component that plays a crucial role in resistance [15,29,47]. However, in this study, the clinical isolate PS-2 was found to be highly resistant due to the coordination of MBL, efflux pump, and biofilm. PS-2 was more resistant to tetracycline and other antibiotics, so this isolate was taken for further study. For any isolate which shows luxuriant growth on the higher concentration of Et-Br, it is supposed that their resistance mechanism is mediated by an efflux pump and production of metallo-β-lactamase makes them be considered as superbugs [5,14,17,39,40,41,48,49,50]. The critical superbug *P. aeruginosa* exploits efflux pump, biofilm, and MBL to achieve a high degree of resistance [5,14,45,47,50,51,52].

Nanomaterials are supposed to be effective warheads to overcome multiple drug resistance [53,54,55,56]. Some of the nanomaterials directly possess antibacterial activity themselves [54,55,57]. However, some of the nanomaterials enhance the antibacterial activity of partner drugs. In this study nanomaterials, GO, GO/ZnO, and other nanomaterials (GO–CS, GO–CS/ZnO, and ZnO) were not efficient antibacterial agents as their MIC was higher; however, in combination, these were able to enhance the activity of ethidium bromide and tetracycline. The reduction in MIC of ethidium bromide and tetracycline indicated that the used nanomaterials were able to inhibit the membrane components of the bacterial cell. Several reports indicated that earlier many nanomaterials were able to potentiate partner drugs many times by inhibiting the transporter proteins [22,23,24,53,54,55,56,57,58,59,60]. In this study, all the used nanomaterials (GO, GO/ZnO, GO–CS, GO–CS/ZnO, and ZnO) were also able to inhibit the biofilm formation. Several reports indicated that nanomaterials were reported to inhibit biofilm formation [47,56,60,61,62,63]. Resistance towards ethidium bromide (Et-Br) is considered a marker of MDR mediated by over expression of efflux pumps [6,48,49,50,51,61,62,64,65]. According to the two newest reports, gold nanorods enhance the activity of ciprofloxacin by inhibiting the biofilms and a silver nanocomposite was found as a resistance reversal agent [66,67]. In this study, nanomaterials were found to synergize the partner drugs by inhibiting the biofilm synthesis/transporter proteins. This will be helpful for the development of nanomaterial-coated antibiotics to overcome the resistance.

## 5. Conclusions

The used nanomaterials may be the powerful weapon the managing the MDR *P. aeruginosa* by (i) reduction of the antibiotic dose; (ii) low concentration of antibiotics may reduce frequency of drug resistance; and (iii) enhancing the effectiveness of antibiotics in combination. These results give an insight into the ways to synthesize novel anti-bacterial agents from nanomaterials.

## Figures and Tables

**Table 1:** Interaction study of nanomaterials with tetracycline against PS-2.

TET + Nanomaterials	MIC (mg/L) Alone Combination Nanomaterials/TET)		FICI + SD	Interaction	Fold Dilution in the MIC of Tetracycline
TET	800	-	-	-	-
GO	800	25/12.5	0.0468 ± 0.03	Synergy	64
GO/ZnO	800	25/12.5	0.0468 ± 0.03	Synergy	64
GO–CS	800	25/50	0.0937 ± 0.05	Synergy	16
GO–CS/ZnO	400	25/50	0.125 ± 0.02	Synergy	16
ZnO	800	25/12.5	0.0468 ± 0.04	Synergy	64

**Table 2:** Interaction study of nanomaterials (GO, GO/ZnO, GO–CS, GO–CS/ZnO, and ZnO) with ethidium bromide against PS-2.

EtBr + Nanomaterials	MIC (mg/L) Alone Combination Nanomaterials/EtBr		FICI + SD	Interaction	Fold Dilution
EtBr	1600	-	-	-	-
GO	800	25/800	0.531 ± 0.05	Additive	2 fold
GO/ZnO	800	25/800	0.531 ± 0.04	Additive	2 fold
GO–CS	800	25/200	0.156 ± 0.02	synergy	8 fold
GO–CS/ZnO	400	25/200	0.187 ± 0.03	Synergy	8 fold
ZnO	800	25/100	0.093 ± 0.01	Synergy	16 fold

**Table 3:** Drug ability study of nanomaterials (GO, GO/ZnO, GO–CS, GO–CS/ZnO, and ZnO).

	Concentration of TET (mg/L)	Concentration of Nanomaterials (mg/L)	Cfu/mL + SD
Tetracycline	100	-	9.6 × 10^10^ ± 0.1
	200	-	7.6 × 10^10^ ± 0.1
	400	-	3.5 × 10^10^ ± 0.05
	800	-	No growth
Tetracycline + GO	100	25	No growth
	200	25	No growth
	400	25	No growth
	800	25	No growth
Tetracycline + GO/ZnO	100	25	No growth
	200	25	No growth
	400	25	No growth
	800	25	No growth
Tetracycline+ GO-CS	100	25	No growth
	200	25	No growth
	400	25	No growth
	800	25	No growth
Tetracycline + GO-CS/ZnO	100	25	No growth
	200	25	No growth
	400	25	No growth
	800	25	No growth
Tetracycline + ZnO	100	25	No growth
	200	25	No growth
	400	25	No growth
	800	25	No growth

## Data Availability

Data sharing not applicable.

## Supplementary Materials

The following are available online at https://www.mdpi.com/article/10.3390/nano12010117/s1, Figure S1: SEM image of GO@CS/ZnO; Table S1: MIC of different antibiotics against PS-2, PS-3 and PS-11.
