# Supplementary material for: Drug Resistance Reversal Potential of Nanoparticles/Nanocomposites via Antibiotic’s Potentiation in Multi Drug Resistant P. aeruginosa
Source: Nanomaterials (Basel). 2021 Dec 30;12(1):117. doi: 10.3390/nano12010117 (PMC8746836; doi:10.3390/nano12010117)
Supplement: Supplementary file 1 [file nanomaterials-12-00117-s001.zip › nanomaterials-1498062 supplementary updated PDF.pdf]

nanomaterials

Supplementary Materials

Drug Resistance Reversal Potential of Nanoparticles/Nanocomposites via Antibiotic's Potentiation in Multi Drug Resistant *P. aeruginosa*

Pratima Pandey [1,2,†], Rajashree Sahoo [3,†], Khusbu Singh [3], Sanghamitra Pati [3], Jose Mathew [1], Avinash Chandra Pandey [2], Rajni Kant [4], Ihn Han [5], Eun-Ha Choi [5,*], Gaurav Raj Dwivedi [4,*] and Dharmendra K. Yadav [6,*]

[1] Department of Biotechnology, Bundelkhand University, Jhansi 284128, India; bajpai.pratima@rediffmail.com (P.P.); jose_feb_25@yahoo.co.in (J.M.);
[2] Nanotechnology Application Centre, University of Allahabad, Allahabad 211002, India; acpandey@iuac.res.in
[3] Microbiology Department, ICMR-Regional Medical Research Centre, Bhubaneshwar 751023, India; rajashree.rainy777@gmail.com (R.S.); singh.khusbu253@gmail.com (K.S.); drsanghamitra12@gmail.com (S.P.);
[4] Microbiology Department, ICMR-Regional Medical Research Centre, Gorakhpur 273013, India; rajnikant.srivastava@gmail.com
[5] Plasma Bioscience Research Center, Applied Plasma Medicine Center, Department of Electrical & Bio-logical Physics, Kwangwoon University, Seoul 01897, Korea; hanihn@kw.ac.kr
[6] College of Pharmacy, Gachon University, Hambakmoeiro 191, Yeonsu-gu, Incheon City 406-799, Korea
* Correspondence: choipdp@gmail.com (E.-H.C.); grdnikhil@gmail.com (G.R.D.); dharmendra30oct@gmail.com (D.K.Y.); Tel.: +82-32-820-4948
† These authors contributed equally to this work

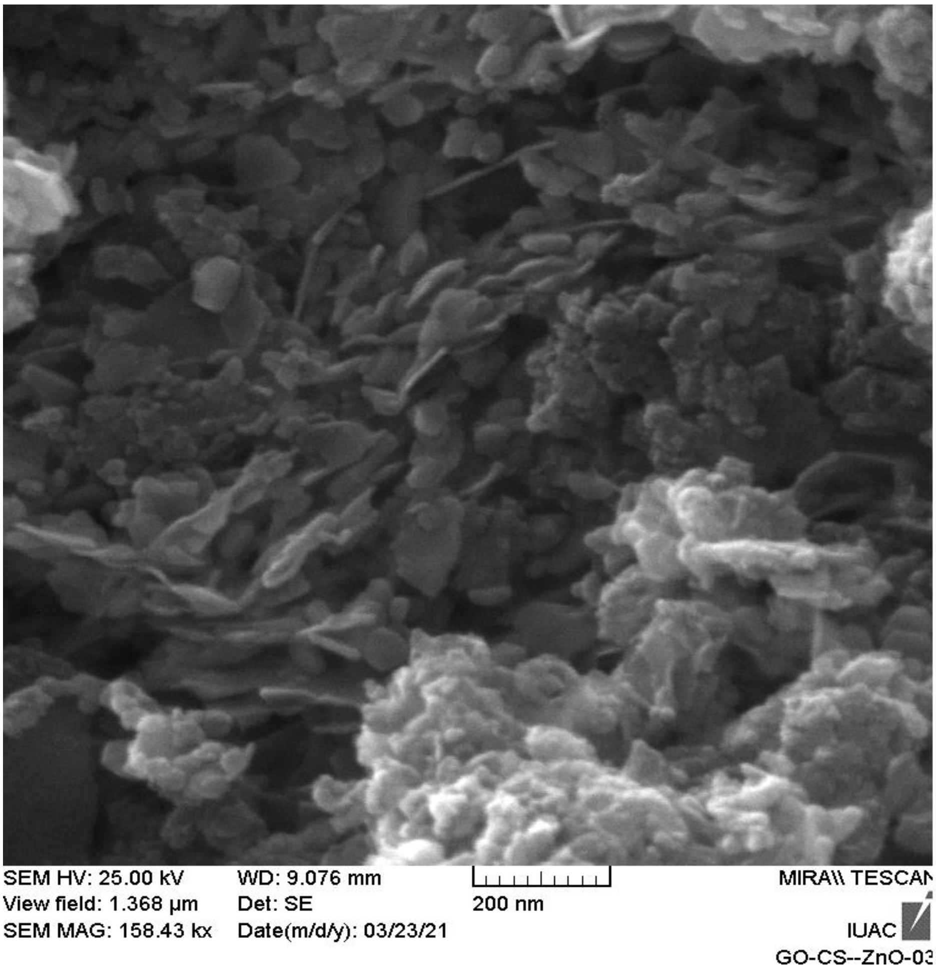

Figure S1. SEM image of GO@CS/ZnO.

Table S1. MIC of different antibiotics against PS-2, PS-3 and PS-11.

SL. No.	Antibiotics	MIC of antibiotics (mg/L) against clinical isolates of *P. aeruginosa*		
		PS-2	PS-3	PS-11
1.	Ampicillin	1600	800	800
2.	Cloramphenicol	1600	1600	400
3.	Rifampicin	800	800	800
4.	Gentamicin	1600	1600	1600
5.	Ceftazidime	1600	1600	1600
6.	Ciprofloxacin	800	400	400
7.	Tetracycline	800	400	400
8.	Sulfamethoxazole	1600	1600	1600
